# The effects of different sensory augmentation on weight-shifting balance exercises in Parkinson’s disease and healthy elderly people: a proof-of-concept study

**DOI:** 10.1186/s12984-015-0064-y

**Published:** 2015-09-02

**Authors:** Beom-Chan Lee, Timothy A. Thrasher, Stanley P. Fisher, Charles S. Layne

**Affiliations:** Department of Health and Human Performance, University of Houston, Houston, TX USA; Center for Neuromotor and Biomechanics Research, University of Houston, Houston, TX USA; Movement Disorders and Neurorehabilitation Center, Houston Methodist Neurological Institute, Houston, TX USA; Center for Neuro-Engineering and Cognitive Science, University of Houston, Houston, TX USA

## Abstract

**Background:**

Earlier versions of biofeedback systems for balance-related applications were intended primarily to provide “alarm” signals about body tilt rather than to guide rehabilitation exercise motion. Additionally, there have been few attempts to evaluate guidance modalities for balance rehabilitation exercises. The purpose of this proof-of-concept study is to evaluate the effects of guidance modalities during common dynamic weight-shifting exercises used in clinical settings.

**Methods:**

A motion guidance system providing visual biofeedback, vibrotactile biofeedback, or both, was used during weight-shifting exercises. Eleven people with idiopathic Parkinson’s disease (PD) and nine healthy elderly people participated. Each participant wore a six-degree-of-freedom inertial measurement unit (IMU) located near the sacrum and four linear vibrating actuators (Tactors) attached to the skin over the front, back, and right and left sides of the abdomen. The IMU measured angular displacements and velocities of body tilt in anterior-posterior (A/P) and medial-lateral (M/L) directions. Participants were instructed to follow a slow moving target by shifting their weight in either the A/P or M/L direction up to 90 % of their limits of stability (LOS). Real-time position error was provided to participants in one of three sensory modalities: visual, vibrotactile, or both. Participants performed 5 trials for each biofeedback modality and movement direction (A/P and M/L) for a total of 30 trials in a random order. To characterize performance, position error was defined as the average absolute difference between the target and participant movements in degrees.

**Results:**

Simultaneous delivery of visual and vibrotactile biofeedback resulted in significantly lower position error compared to either visual or vibrotactile biofeedback alone regardless of the movement direction for both participant cohorts. The pairwise comparisons were not significantly different between visual and vibrotactile biofeedback.

**Conclusion:**

The study is the first attempt to assess the effects of guidance modalities on common balance rehabilitation exercises in people with PD and healthy elderly people. The results suggest that combined visual and vibrotactile biofeedback can improve volitional responses during postural tracking tasks.

Index Terms – sensory augmentation, weight-shifting balance exercise, guidance modality, vibrotactile biofeedback, visual biofeedback, Parkinson’s disease.

## Background

Parkinson’s disease (PD) affects at least 10,000,000 people worldwide [1]. The cardinal motor systems are tremor, bradykinesia, rigidity, and postural instability [2]. Dopaminergic medication and surgical treatment (e.g., deep brain stimulation) have been shown to suppress the symptoms of tremor, bradykinesia, and muscle rigidity [3–6], but does not prevent the progression of the disease [7]. The treatments are not as effective in treating postural instability [8, 9], which increases loss of balance [10] and risk of falling [11], thus restricting motor performance and reducing the level of independence in daily activities [12].

Several studies have shown that physical and balance rehabilitation regimens can improve postural stability in people with PD for short (hours to days) and long (weeks to months) periods [13–17]. Recently, Smania et al. [13] evaluated the effect of intensive balance training sessions on postural stability in 64 people with idiopathic PD during conventional balance training (e.g., dynamic weight-shifting balance exercise and destabilization of the body’s center of mass (COM)) under the supervision of a physical therapist. The results demonstrated that postural stability and confidence improved and fall events decreased after balance training was completed, and that the training effects lasted for at least 1 month. However, many balance-impaired people with PD cannot perform clinical balance training regimes due to cost, limited availability of physical therapists [18, 19], or memory loss [20, 21]. Moreover, when given exercises to practice at home, compliance generally decreases over time due to the absence of real-time feedback [22–24]. For these and other reasons, there is growing interest in assistive device design and development with sensory augmentation.

Sensory augmentation, popularly called biofeedback, is a technique of augmenting or replacing compromised sensory information through external cues to facilitate the retraining of sensorimotor functions (see [25] for review) during rehabilitation. Utilizing visual [26, 27], auditory [28, 29], or tactile cues [30–35], biofeedback technologies provide additional information about body motion. Among the aforementioned biofeedback modalities, vibrotactile information as a means of touch input can be used to mimic the role of a therapist’s hands by providing somatosensory information for movement corrections [36–38]. A number of studies have demonstrated that wearable vibrotactile biofeedback systems improve balance performance in anterior-posterior (A/P) and medial-lateral (M/L) directions in people with vestibular deficits [31, 32, 39], older adults [30, 33, 39] and people with PD [34, 40]. The results showed that improved postural performance was observed over short periods of time (hours to days) after a small number of sessions (e.g., two sessions, 3 h each) with real-time vibrotactile balance aids.

Most vibrotactile biofeedback systems are designed to send “alarm” signals about body tilt rather than to provide guidance for correction of performance errors while performing exercises. In addition, the effects of guidance modality on balance-related exercises have not yet been studied (e.g., whether one biofeedback modality is more effective than another, combining visual and vibrotactile biofeedback to leverage voluntary motor control during rehabilitation exercises, etc.).

Motivated by the need to improve clinical and in-home exercise regimens, we propose a wearable guidance system for dynamic weight-shifting exercises. We 1) describe the system design to convey guidance information for dynamic weight-shifting exercises and 2) quantitatively assess the effects of guidance modalities (visual vs. vibrotactile vs. simultaneous visual and vibrotactile biofeedback) during dynamic weight-shifting exercises in PD and healthy elderly people. The eventual goal is to design a system for use by balanced-impaired people performing therapist-assigned exercises in a clinic, at home, or in an environment with limited access to balance therapy.

## Methods

### Wearable guidance system for dynamic weight-shifting exercises

**Fig. 1:**
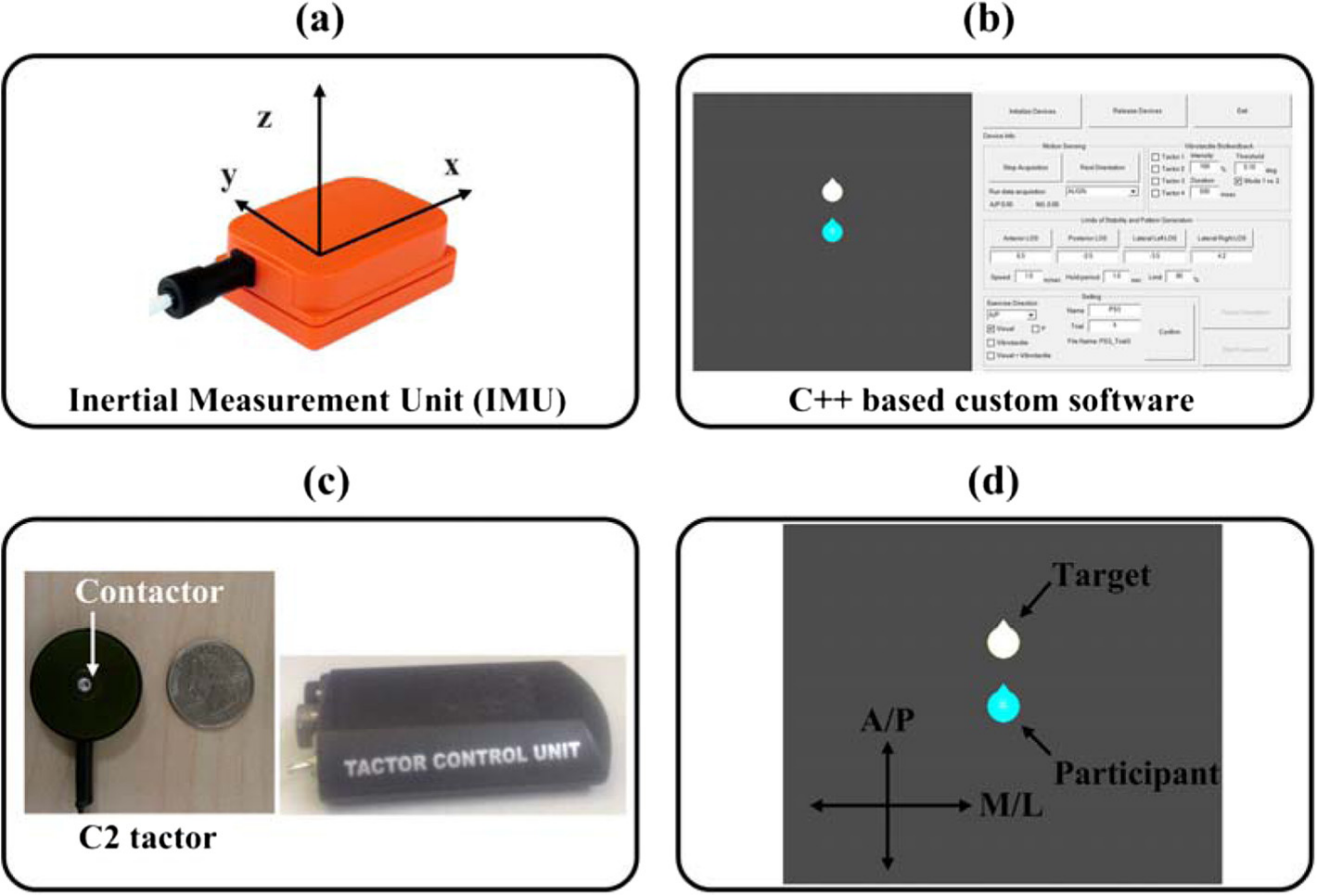
A system configuration. **a** Sensing system. **b** Custom software. **c** C2 tactor and tactor control unit. **d** Visual biofeedback. A white and light blue object depicts the target and participant’s movements in A/P and M/L directions

**Fig. 2:**
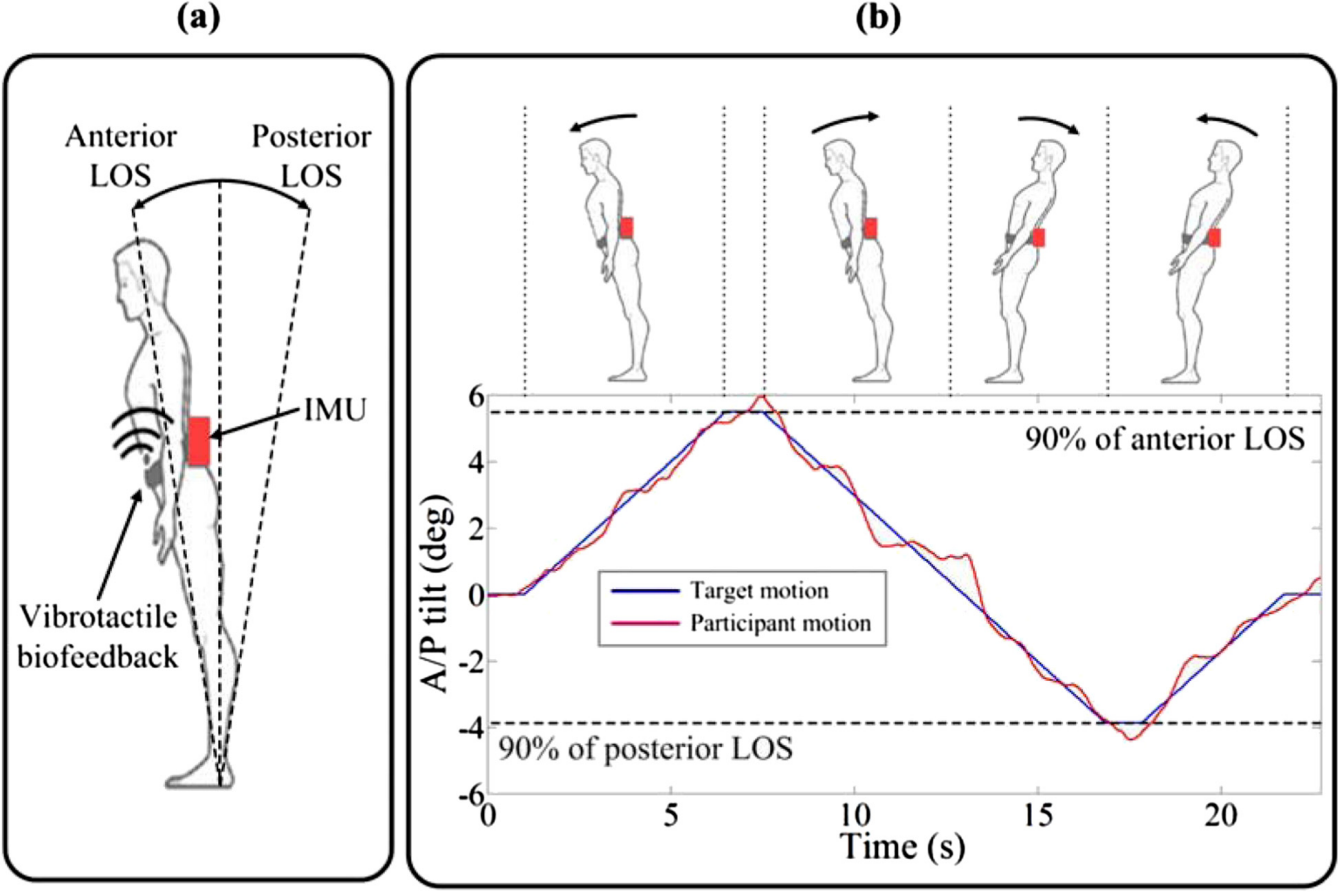
**a** Sensor and tactor location. **b** Representative sample data from one participant with PD during the A/P dynamic weight-shifting exercise. Images shown in the top panel indicate movement directions corresponding to the movement trajectories shown in the bottom panel. Solid blue and red lines shown in the bottom panel represent the target motion (generated by custom software after measuring the participant’s 90 % of LOS in the A/P direction) and participant’s motion, respectively. Dashed black lines represent the participant’s 90 % of LOS in both the anterior and posterior directions

Figure 1 illustrates the key components of the biofeedback system: a commercial six degree-of-freedom inertial measurement unit (IMU; Xsens Technologies, NL), custom software, vibrotactile control circuit and four C2 tactors (Engineering Acoustics Inc., Casselberry, FL, USA), and a virtual environment for displaying visual biofeedback. The IMU measured angular displacements and velocities in the A/P and M/L directions. Manufacturer specifications indicated a static accuracy better than 0.5° and an angular resolution equalling 0.05° (Xsens Technologies, NL). IMU signals were sampled at a rate of 100 Hz. The tactor driving circuit (Fig. 1 (c)) generated sinusoidal signals to actuate the C2 tactors at a frequency of 250 Hz and with peak-to-peak amplitude of 200 μm [41]. The C2 tactor is a linear actuator with a cylindrical moving contactor oscillating perpendicular to the skin at the center with a cross-sectional area of 58.6 mm^2^. The IMU and tactors were attached with Velcro to an elastic belt worn around the torso, as shown in Fig. 2 (a). The IMU was placed on the lower back at approximately the level of the L5/S1 vertebra corresponding to the body’s COM. Four tactors were placed on the skin over the front, back, and right and left sides of the torso approximately at the level of the L4/L5 vertebra.

Custom software that was implemented using Microsoft Visual C++ generated the target movement trajectories in degrees by measuring the participant’s 90 % of limits of stability (LOS) in A/P and M/L directions, as illustrated in Fig. 2(b). Typically, the LOS has been used to indicate the stable area in A/P and M/L directions over which people can move their COM and maintain postural equilibrium without changing their base of support. Movement speed was set to 1 °/s, a preferred speed for dynamic weight-shifting exercises in a clinical setting [42, 43]. A proportional plus derivative control signal activated the tactors based on differences in both body tilt angle and angular velocity between the target and participant’s motions [44]:

1 $$ Error\kern0.28em signal=\left({\theta}_{\mathrm{target}}-{\theta}_{\mathrm{participant}}\right)+{K}_d\left({\dot{\theta}}_{\mathrm{target}}-{\dot{\theta}}_{\mathrm{participant}}\right) $$

where θ and $\dot{\uptheta}$ represented the body tilt angle in degrees and the angular velocity of body tilt in °/ms, respectively. K_d_was set to 0.5 ms based on a previous study [44]. Custom software provided command signals to activate tactors when the absolute value of the error signal exceeded the tactor activation threshold set at 1.0° [44]. Vibrotactile biofeedback was deactivated when the error signal dropped below 1.0°, and thus the tactor activation was binary in nature (either on or off).

Similar to a computerized visual display of body sway, two virtual objects were displayed in a virtual environment in order to indicate target and participant’s movements, as illustrated in Fig. 1(d). A white and light blue object moved according to the target movements generated by custom software and participant’s movement measured by the IMU (i.e., body tilt with respect to the base of support), respectively. For instance, A/P motion of the measured participant’s body tilt was continuously displayed as upward and downward motion of the virtual object (i.e., light blue object), and M/L motion of the measured participant’s body tilt was continuously displayed as left and right motion of the virtual object (i.e., light blue object). Likewise, the virtual object (i.e., white object) was continuously moved in the A/P or M/L direction associated with generated movement trajectories of the target. Each virtual object was continuously displayed on a 52 inch monitor at an update rate of 30 Hz [45].

### Participants

Eleven idiopathic people with PD (70.0 ± 8.1 year; 2 females, 9 males), referred to as the “PD group”, having bilateral symptoms with impaired postural stability (i.e., a score of 3 or 4 on the Hoehn and Yahr scale [46]) and nine healthy elderly (67.8 ± 6.6 years; 7 females, 1 males) spouses of the people with PD, referred to as the “control group”, participated. All participants were naïve to the purpose of the experiment.

Potential participants (recruited from a movement disorders clinic at the Methodist Neurological Institute, Houston, Texas) were excluded if they: 1) could not read and comprehend English; 2) had difficulty standing for prolonged periods (e.g., 10 min); 3) were unable to stand for 1 min with their eyes open and closed; 4) had severe distal sensory loss as demonstrated by a 5.07 g monofilament test (i.e., potential participants who were unable to report a sensation of the monofilament more than three out of four times on each foot (plantar surface of each great toe and plantar surfaces of the 1st, 3rd, and 5th metatarsal heads of each foot) were excluded); 5) had limited ankle range of motion, demonstrated ankle dorsiflexor/plantar flexor weakness, or great toe weakness; 6) reported lower extremity fracture/sprain in the past six months or previous lower extremity total joint replacement; 7) were medically unstable (chest pain upon exertion, dyspnea); 8) had active motion-provoked vertigo or a diagnosed vestibular deficit; or 9) had a cognitive level less than 24 determined by the Mini Mental State Examination (MMSE) [47].

Each participant provided informed consent prior to the start of the experimental procedures. The study was conducted in accordance with the Helsinki Declaration and approved by the Committee for the Protection of Human Subjects at the University of Houston.

### Procedure

All tests were conducted with people who had PD and were taking medication to alleviate tremor, bradykinesia, and muscle rigidity. Prior to beginning the experimental session, participants’ balance performance was assessed by the Sensory Organization Test (SOT) using a Balance Master® (Balance Master®; NeuroCom, USA). The SOT, which is commonly used to quantitatively assess the sensory and voluntary motor control of balance during standing, measures postural sway with two force plates in response to visual and mechanical (moving platform) perturbations [48, 49]. During the SOT, a safety harness was used for all participants. Each of the six sensory conditions in the SOT was randomly provided three times for a total of 18 trials. Each trial was 20 s in duration with 20 s of rest between trials. The results of the SOT were used to evaluate baseline balance performance for the two groups.

At the beginning of the experimental session, all participants were instrumented with the IMU and four tactors. Their LOS in both A/P and M/L directions were obtained from body movements in degrees that corresponded to the furthest deviations of the body tilt in each direction from a neutral starting point. All participants performed 12 familiarization trials (i.e., 3 modalities × 2 directions × 2 repetitions) to acclimate to the guidance modalities (visual, vibrotactile, and simultaneous visual and vibrotactile biofeedback) during dynamic weight-shifting balance exercises. After the completion of the familiarization trials, all participants were provided a 5 min seated rest. During the experimental session, all participants performed dynamic weight-shifting balance exercises as a function of the modality and direction with 5 repetitions for a total of 30 trials (i.e., 3 modalities × 2 directions × 5 repetitions). The order of trials was randomized for each participant.

During all familiarization and experimental trials, participants were asked to stand on a firm surface with their arms held down at their sides and their feet hip-width apart. They were instructed to move their bodies by locking knees and hip joints (e.g., behaving as inverted pendulums) during dynamic weight-shifting balance exercises. During trials involving vibrotactile biofeedback, they were instructed to move the body in the direction opposite the vibration until the vibration stopped. For visual guidance (i.e., trials with visual or simultaneous visual and vibrotactile biofeedback), they performed exercises by looking at two virtual objects representing their actual movements and target movements displayed on a 52 inch monitor placed approximately 2 m ahead at eye level. The duration of each trial was less than 45 s. Consecutive trials were separated by approximately a 20 s rest period. All participants were provided a 5 min seated rest after every 10 trials.

Following the completion of the experimental trials, participants’ LOS in both A/P and M/L direction were re-measured to evaluate the range of motion. After each experimental trial, custom software stored the dependent measures of target and participant angular displacements and the two sets of LOS values in text format for analysis.

### Data analysis

MATLAB (The MathWorks, Natick, MA) was used to process recorded data. A SOT score and range of LOS in both A/P and M/L directions were used to evaluate participants’ baseline balance performance and the effects of dynamic weight-shifting balance exercises, respectively. The composite SOT score ranged between 0 and 100%. No movement resulted in a score of 100, whereas a fall resulted in a score of 0. To characterize participants’ ability to perform dynamic weight-shifting balance exercises as a function of the guidance modality and movement direction, position error was defined as the average absolute difference between the target and participant movements in degrees.

Levene’s test of equality of error variances verified that three metrics (SOT score, range of LOS, and position error) were normally distributed. An analysis of variance (ANOVA) assessed the main and interaction effects for all metrics. A one-way ANOVA assessed the main effects of the group (PD and control) for the SOT score. For the range of LOS, a three-way ANOVA assessed the main effect of the group, exercise (pre- and post-exercise), and direction (A/P and M/L) as well as their interactions. For the position error, five repetitions of each trial were averaged for each participant because the effect of repetition was not significant as determined by a repeated measures ANOVA. Therefore, the three-way ANOVA assessed the main effects of the group, modality (visual, vibrotactile, and combined visual and vibrotactile biofeedback), and direction as well as their interactions. Post hoc analysis was performed using Sidak’s method to determine the factors influencing the main and interaction effects. The level of significance was set at *p* < 0.05.

## Results

**Fig. 3:**
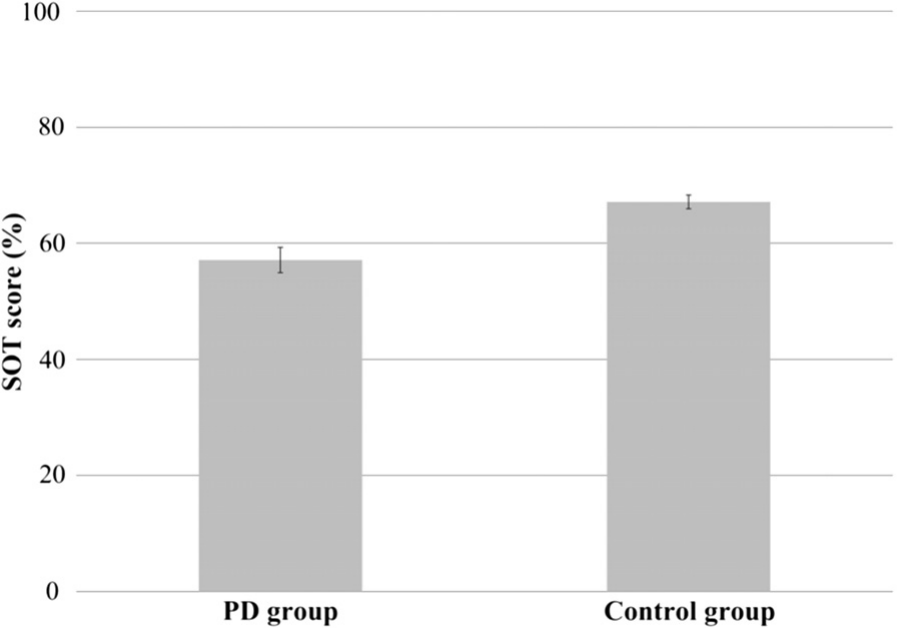
Average SOT scores for the PD (*n* = 11) and control (*n* = 9) group

Figure 3 illustrates the mean values of SOT scores for each group. No significant differences as a function of the group were observed in the SOT scores [F(1, 17) = 1.24, *p* = 0.28]; the PD group had a lower score (57.1) than the control group (67.1).

**Fig. 4:**
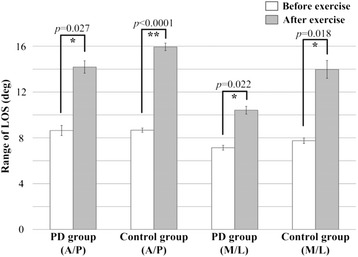
Average range of LOS in the A/P and M/L direction as a function of the group before and after dynamic weight-shifting balance exercises guided by biofeedback. Error bars indicate standard error of the corresponding average (* *p* < 0.05, ** *p* < 0.0001)

Figure 4 shows the mean range of LOS as a function of the direction (i.e., A/P and M/L) for each group. Table 1 summarizes the results of the statistical analysis for the range of LOS. The three-way ANOVA indicated significant main effects of the exercise (*p* < 0.001) and direction (*p* < 0.05), whereas the group and interaction effects were not significant (*p* > 0.05). Indeed, the range of participants’ LOS in both A/P and M/L directions improved for both groups after the completion of the 30 experimental trials. For instance, the range of LOS corresponding to the A/P direction increased by 64.17 % in the PD group and 83.85 % in the control group. Similarly, the range of LOS corresponding to the M/L direction increased by 45.90 % in the PD group and 80.32 % in the control group.

**Table 1:** Statistical analysis results of the range of LOS for group (G), exercise (E), and direction (D) and their interaction

Dependent variable	Effects	DF	F value	Pr > F
Range of LOS	G	1, 68	2.43	0.124
	E	1, 68	33.97	<0.0001^a^
	D	1, 68	4.54	0.037^a^
	G × E	1, 68	1.49	0.266
	G × D	1, 68	0.39	0.535
	E × D	1, 68	0.75	0.390
	G × E × D	1, 68	0.10	0.751

^a^Statistical significance

**Fig. 5:**
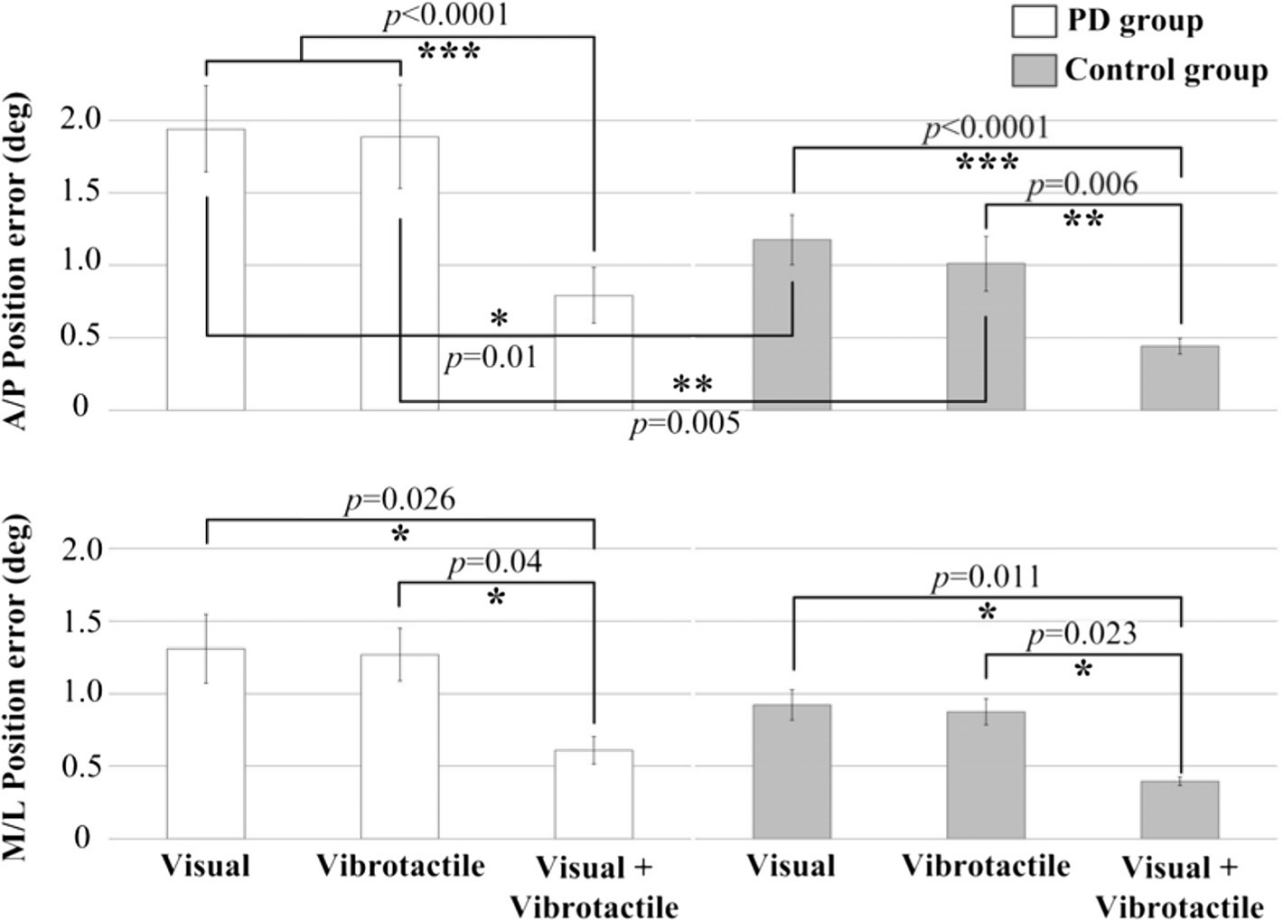
Average position error as a function of the direction, modality, and group. Error bars indicate standard error of the corresponding average (* *p* < 0.05, (** *p* < 0.001, ** *p* < 0.0001)

Table 2 shows that the three-way ANOVA applied to the position error indicated significant main effects of the group and modality, whereas the direction and interaction effects were not significant (*p* > 0.05). Figure 5 depicts the position error between the target and participant movements in both directions. A post hoc analysis showed that all participants had the smallest position error when they performed exercises with combined visual and vibrotactile biofeedback regardless of direction, and that the control group had a smaller position error than the PD group when they performed exercises with either visual or vibrotactile biofeedback in the A/P direction. Other pairwise comparisons between visual and vibrotactile biofeedback were not significant regardless of group and direction.

**Table 2:** Statistical analysis results of the cross-correlation and position error for group (G), modality (M), and direction (D) and their interaction

Dependent variable	Effects	DF	F Value	Pr > F
Cross-correlation	G	1102	29.39	<0.0001^a^
	M	2102	27.16	<0.0001^a^
	D	1102	0.18	0.676
	G × M	2102	2.79	0.126
	G × D	1102	0.92	0.341
	M × D	2102	0.38	0.689
	G × M × D	2102	1.08	0.343
Position error	G	1102	16.20	<0.0001^a^
	M	2102	18.42	<0.0001^a^
	D	1102	1.92	0.110
	G × M	2102	0.85	0.432
	G × D	1102	1.85	0.176
	M × D	2102	0.78	0.462
	G × M × D	2102	0.20	0.822

^a^Statistical Significance

## Discussion

Our key finding is that both groups had the smallest position error between the target and participant movements when performing weight-shifting balance exercises accompanied by simultaneous delivery of visual and vibrotactile biofeedback regardless of A/P and M/L directions; the increased range of LOS in both directions occurred after the completion of 30 experimental trials. The finding aligns with recent studies noting that participants significantly reduce position errors when mimicking the simple task of slowly bending at the waist was combined with receiving visual and vibrotactile biofeedback [50], and that vibrotactile biofeedback enhances participants’ performance (dancing) more than video instruction only [51].

It is generally accepted that sensory augmentation (biofeedback) provided by a technical display through a visual, auditory, or tactile channel can enhance motor learning and re-learning during rehabilitation [25]. Recent research has reported that multimodal biofeedback can facilitate motor learning more than unimodal biofeedback (see [52] for an overview). Also, there is evidence that simultaneous delivery of visual and vibrotactile biofeedback can be effective for spatiotemporal learning during motor tracking tasks [52]. Moreover, the positive effects of concurrent multimodal biofeedback during motor learning tasks have been described as being facilitated by intersensory facilitation [53] and increased cellular activity in the primary sensorimotor cortex [53]. Hence, it is speculated that the proposed system in this study would facilitate dynamic movement coordination when participants in both groups performed balance exercises with multimodal biofeedback.

Research in the field of motor learning with biofeedback has extensively investigated visual information in the context of optimizing biofeedback (see [52] for an overview), given that vision dominates other sensory channels, at least for perceiving spatial information [54]. The quasi-identical results between visual and vibrotactile biofeedback in this study suggest that vibrotactile biofeedback providing temporal aspects of the target motion can allow participants to correct their movement errors. This is in line with previous findings that touch guidance facilitates movement timing in targeting tasks [55, 56]. In the context of motion guidance, therefore, vibrotactile can provide spatial properties of the movement [57].

We also found that the control group produced significantly better motion replication than the PD group with only visual or only vibrotactile biofeedback in the A/P direction. We attribute the finding to the greater magnitude of A/P sway (i.e., the greater magnitude of A/P sway can result in less accurate movement coordination in people with PD [8–11]). Less accurate movement coordination in the A/P direction also can be associated with the slowness in movement initiation (slow reaction time and movement time) in people with PD compared to movement initiation in healthy people [58]. However, we observed no significant group effects with combined visual and vibrotactile biofeedback in A/P and M/L directions. It can be interpreted that multimodal biofeedback facilitates movement initiation more effectively than unimodal (e.g., visual [59], auditory [60], or proprioceptive [61]) biofeedback in people with PD, which confirms that the two groups in this study benefitted equally from simultaneous visual and vibrotactile biofeedback.

No significant differences of the SOT scores between the two groups were observed in the evaluation of baseline balance performance. This finding may be attributed to the differences in the gender distributions of the groups. Multiple studies, however, have reported no gender differences in balance parameters (e.g., postural sway, sway area, sway path length, etc.) [62–65] and equilibrium quotient scores on SOT 1–4 [66] in elderly populations. Presumably, the quasi-identical SOT scores between the people with PD and healthy elderly people observed in this study can be related to postural inflexibility (i.e., increased stiffness) in people with PD resulting in small postural sway as observed by Horak et al. [67]. Indeed, our results and assumption are in line with recent findings that overall SOT scores did not differ between people with PD in the on phase of the medication cycle and healthy elderly people [68, 69].

Nevertheless, our study revealed that the two groups significantly improved their range of LOS after performing a single session of 30 trials with dynamic weight-shifting balance exercises guided by biofeedback. The result confirms previous findings that dynamic balance exercises involving shifting the body’s COM smoothly and rhythmically improves balance performance in people with a high risk of falling [42, 43].

The limitations of this proof-of-concept study are a relatively small sample size, and an imbalanced gender distribution within and between the groups. Despite the limitations, the results confirm that combined visual and vibrotactile biofeedback can improve volitional responses during postural tracking tasks. In addition, dynamic weight-shifting exercises guided by biofeedback lead to postural stability improvements in people with PD and elderly people.

## Conclusion

This study describes the effects of guidance modalities during dynamic weight-shifting exercises, which are commonly used in balance rehabilitation, in people with PD and healthy elderly people. The results demonstrated the superiority of simultaneous delivery of visual and vibrotactile biofeedback over unimodal biofeedback (i.e., visual or vibrotactile biofeedback) regardless of participant cohort. A small number of biofeedback guided dynamic weight-shifting balance exercises resulted in the increased range of LOS in both A/P and M/L directions after removing the biofeedback. We conclude that wearable technologies incorporating visual and vibrotactile feedback can assist in performing exercise regimens, potentially improving the overall quality of life for people with PD and older adults.

Although recent advances in assistive technologies with biofeedback have offered advantages for balance rehabilitation in balance-impaired people (e.g., older adults [30, 39], vestibular deficits [32, 39], and people with PD [27, 29, 35]), to date no technologies are readily available for use in clinical and/or home-based settings. Future research will explore multimodal biofeedback in a smartphone-based system [31] to be used by balance-impaired people when performing clinic and in-home rehabilitation and exercise regimens. To further understand the context in which smartphone-based balance rehabilitation aids combined with multimodal biofeedback can lead to steady improvements in balance performance for people with PD, future research will conduct a longitudinal study with an increased sample size, aged matched controls, and greater balance across genders. Our eventual goal is to design a system for in-home use that can augment balance rehabilitation exercises and help to reduce healthcare costs associated with therapeutic balance rehabilitation programs for balance-impaired people.

## Abbreviations

ANOVA: Analysis of variance
A/P: Anterior-posterior
COM: Center of mass
IMU: Inertial measurement unit
LOS: Limits of stability
M/L: Medial-lateral
MMSE: Mini mental state examination
SOT: Sensory organization test
PD: Parkinson’s disease.
